# Molecular epidemiology of influenza, respiratory syncytial virus, SARS-CoV-2, other respiratory viruses and bacteria among children 0–2-year-olds in West Bengal: a one-year influenza-like illness surveillance study (2022–2023)

**DOI:** 10.3389/fepid.2025.1578951

**Published:** 2025-05-20

**Authors:** Tila Khan, Sayantan Halder, Ranjan Saurav Das, Abhishek Jaiswal, Pearl Helena Scott Leo, Arabinda Mahato, Tarapada Ghosh, Parthasarathi Satpathi, Sangeeta Das Bhattacharya

**Affiliations:** ^1^ Global Health Research Laboratory, School of Medical Science & Technology, Indian Institute of Technology Kharagpur, Kharagpur, India; ^2^ Viral Research and Diagnostic Laboratory, Department of Microbiology, Midnapore Medical College & Hospital, Midnapore, India; ^3^ Paediatrics, Kharagpur Sub Divisional Hospital, Kharagpur, India; ^4^ Department of Paediatrics and Neonatology, Midnapore Medical College & Hospital, Midnapore, India; ^5^ Section of Internal Medicine and Pediatrics, Christiana Care Health System, Newark, DE, United States

**Keywords:** child, infant, influenza, respiratory syncytial virus, SARS-CoV-2, epidemiology, respiratory tract infections, influenza-like illness

## Abstract

**Background:**

Lower respiratory infections are the leading cause of paediatric morbidity and mortality. This study documents the incidence and etiology of influenza-like illness (ILI) among young children in rural eastern India.

**Methods:**

We conducted a surveillance of ILI in children visiting paediatric clinics of two hospitals in District West Midnapore, West Bengal from April 1, 2022 to March 31, 2023. Nasopharyngeal swabs were collected from children 0–2 years of age with ILI and tested for influenza, respiratory syncytial virus (RSV), and SARS-CoV-2; a representative set for the respiratory panel.

**Results:**

Of 9,923 outpatient children 0-14 years of age screened, 1,001 had ILI, of which 439 (44%) were in 0–2-year-olds. The ILI incidence was 439/4,310 [10.2% (95% CI: 9.29–11.1)] in the 0-2-year-olds, 288/2,473 [11.6% (10.4–12.9)] in >2-5-year-olds, and, 274/3,140 [8.7% (7.7–9.7)] in >5-14-year-olds. Of 390 enrolled children (median age: 12 months), viruses were identified in 23.3%, occurring singly (15%) or with other viruses (1.3%). RSV was the most common virus (12.6%), followed by influenza (6.6%) and SARS-CoV-2 (0.77%). Influenza subtypes included IA/H3 (50%), IA/H1N1pdm2009 (34.6%) and IB (15.4%). IA/H1N1pdm09 predominated during the 2022 monsoon, RSV during 2022 autumn and A/H3 and B during 2023 winters. Cough and difficulty breathing were associated with RSV. The major bacteria detected were *Streptococcus pneumoniae* (55.5%), *Haemophilus influenzae* (29%) and *Moraxella catarrhalis* (3.7%). Other viruses were parainfluenza virus 3 (4.4%), bocavirus (3.7%) and adenovirus (3%). Viral-bacterial co-detections were frequent (20%). Seventeen children required hospitalization, with difficulty breathing increasing hospitalization risk (OR = 4.47, 95% CI: 1.67–12). Children with RSV had increased odds of hospitalization (OR = 3.11, 95% CI: 1–9.26).

**Conclusions:**

The majority of ILI was observed in children aged 0-2 years, with RSV and influenza as major viral causes associated with ILI. RSV increased the risk of hospitalization. These findings contribute to building the evidence base for maternal RSV immunization policy in India.

## 1 Introduction

Lower respiratory infections (LRI) are the leading infectious cause of illness and deaths worldwide, with *Streptococcus pneumoniae* or pneumococcus as the major contributor (1–3). Influenza and respiratory syncytial virus (RSV) are the most common viral causes, particularly affecting children (4). Infants under six months and pregnant women are vulnerable for severe influenza complications (5, 6). In 2018, there were an estimated 109·5 million influenza virus episodes, 10.1 million influenza-associated LRI cases globally among children under-5, and, 15,300 in-hospital deaths (7). Over one-third of these deaths were in infants under 6 months (36%), and 82% in low-income (LIC) and lower-middle-income countries, LMICs (2, 7). India reported influenza in 11.2% of acute respiratory infections (ARI) among under-fives, contributing to 16–18 million cases and 27,825 deaths (8).

RSV is a significant contributor of bronchiolitis and pneumonia, leading to hospitalizations and deaths among young children worldwide. Nearly all children contract RSV by age two, but the greatest severity is in infants under six months (9, 10). In 2019, RSV caused 3·6 million hospitalizations among children 0-5 years globally, 36% occurring in infants aged 0-6 months (10). The greatest RSV mortality was in infants aged 0–6 months (51%), and LMICs (97%) (10).

Respiratory infections in children often involve multiple pathogens. Secondary bacterial pneumonia caused by *Streptococcus pneumoniae*, *Haemophilus influenzae*, *Staphylococcus aureus* and *Streptococcus pyogenes* following influenza infections cause increased disease severity, and mortality (11, 12). Similarly, RSV infections in hospitalized children are associated with bacterial co-infections (13). However, bacterial presence in the nasopharynx does not always correlate with severity, as asymptomatic carriage is common in children.

The corona virus disease 19 (COVID-19) caused by severe acute respiratory virus syndrome coronavirus 2, SARS-CoV-2, became a global health emergency in January 2020. Compared to adults, SARS-CoV-2 causes less severe illness and deaths among children (14). By July 2022, the World Health Organization reported that children under-five accounted for 2.47% of global COVID-19 cases, and, 0.11% of deaths (15).

Influenza epidemics vary by climate. In temperate climates, influenza occurs during cooler periods with lower humidity, and in tropical and subtropical regions, influenza appears year-round or during high humidity months (16). India's diverse topography and climatic leads to dynamic influenza seasonality at sub-regional levels (17), with peaks during monsoon and winters. RSV epidemics begin in late summer in the tropics, and in winter in temperate regions (18).

During the COVID-19 pandemic, viral interference and non-pharmaceutical measures such as social distancing, travel restrictions, school and community closures, and face mask use, led to a global decline in the transmission of other viruses, including influenza and RSV (19–21). As the pandemic receded and these measures were eased, influenza and RSV cases re-emerged (22–24). India implemented a nationwide lockdown in March 2020 (25) till summer, followed by lifting of restrictions, with influenza activity significantly reduced during 2020–2021 (26). RSV declined in 2020 and increased in 2021 in Western India (27).

In LMICs, limited surveillance and laboratory capacity for respiratory virus detection lead to under-reporting of disease and deaths, hindering prioritization of high-risk groups. Low availability of affordable and reliable diagnostic kits further restricts routine testing (28). Evidence on the disease burden in infants and children is required to guide treatment and inform immunization policy.

The epidemiology of respiratory pathogens associated with influenza-like illness (ILI) among children is less understood in District West Midnapore, West Bengal. Influenza-like illness (ILI) is an indicator of influenza activity (29). Several studies have analysed the incidence of influenza and RSV in different parts of India through ILI or acute respiratory illness surveillance (30–32). However, most of the studies have been focused on children under 5 years and confined to the cities. Our study addressed the need for risk prioritization to build the evidence base for maternal influenza and RSV immunization and paediatric influenza immunization, as they are not part of India's Universal Immunization Program. From April 1, 2022–March 31, 2023, we conducted a systematic hospital-based one-year surveillance study of medically attended ILI among children aged 0–2 years to estimate the proportion of influenza, RSV, SARS-CoV-2, and other respiratory pathogens, virus seasonality, and co-infection patterns.

## 2 Methods

### 2.1 Study setting

West Midnapore, the second largest district in West Bengal's south, consists of three subdivisions: Medinipur Sadar, Kharagpur, and Ghatal. It has four government secondary hospitals and one tertiary hospital, offering primary care, admission, laboratory examinations and medicines for free within the public health system. For ILI, patients typically visit local public or private facilities, though some in rural areas consult unqualified rural medical practitioners. No health utilization survey has been conducted in the district to determine the percentage of population seeking public vs. private health institutions. During the pandemic, special clinics were arranged for respiratory infections, with arrangements for rapid antigen and for molecular SARS-CoV-2 tests at the zonal Viral Research Diagnostic Laboratory (VRDL) in Midnapore Medical College & Hospital (MMCH).

The study was based in two hospital sites viz., Midnapore Medical College & Hospital (MMCH, 22.4217° N, 87.3231° E) and Kharagpur Sub-Divisional Hospital (KSDH, 22.3270° N, 87.3148° E). KSDH, located in Kharagpur, serves a population of 2.47 million in the Kharagpur Sub-Division catchment, 15.6 km away from MMCH. MMCH, located in Midnapore, is the region's only public tertiary care center, that receives referrals from the entire district and adjoining districts. Both sites serve a population of 191,430 children under-2.

### 2.2 Study design

This is a descriptive, observational study that aims to study trends, and patterns of respiratory viruses among children 0–2 years of age to inform public health planning and policy.

### 2.3 Participant enrollment, specimen collection and ILI case definition

Data collection forms were developed and standardized in discussion with the paediatricians, microbiologists, scientists and social workers and translated into Bengali. From April 1, 2022 to March 31, 2023, a systematic hospital-based active ILI surveillance was conducted in the paediatric outpatient departments of MMCH (April 1, 2022–March 31, 2023) and KSDH (September 19, 2022–March 31, 2023). Children two years and under, meeting the case definition of influenza-like illness (ILI) were enrolled. ILI was defined as the sudden onset of measured fever (>38°C) or history of tactile temperature and cough or sore throat or rhinorrhea in the past 3 days, as per the 2008 WHO guidelines, with minor modifications (29, 33). The 2008 definition had ‘absence of another diagnosis’ criterion which we omitted to prevent the exclusion of ILI cases with underlying conditions such as asthma and chronic cardiac disease, that can increase the risk of influenza. The definition required measured “fever (>38°C)”, while we used tactile temperatures history, as home temperature readings were unavailable. Subjects with symptom onset beyond three days were excluded. Sampling targeted ten random specimens per week, across 52 weeks. Although, in only one clinic we found more than ten eligible cases. Therefore, to meet the sample target we approached all eligible cases and sampled those whose guardians’ provided consents.

The study staff visited MMCH clinics twice weekly, usually on Tuesdays and Fridays excluding weekends and holidays, from April 1 (Influenza week 13) to September 18, 2022 (Influenza week 38), except in July COVID-19 wave (once weekly). From September 19 onwards, clinics were planned weekly per site (MMCH: Tuesday/Friday; KSDH: Monday/Wednesday). All eligible cases were enrolled per clinic with an average of 4 ILI cases daily. After written informed consent process with the guardians of children by a trained social scientist, sociodemographic data and clinical data were recorded on data collection forms. A trained laboratory technician collected nylon flocked nasal or nasopharyngeal swabs (Himedia) in 3 ml of viral transport medium (Himedia, MS2760A), stored in cold ice packs immediately in a cool box, and transported them at 4°C within two hours, for storage of two aliquots at −80°C in VRDL, MMCH.

### 2.4 Laboratory testing

All study samples collected were tested by multiplex real-time reverse transcriptase polymerase chain reaction (rRT-PCR), in a batch, for influenza, RSV and SARS-CoV-2 at VRDL, MMCH. The laboratory staff responsible for testing was blinded to the participant's data. RNA was extracted manually (HiPurA Viral RNA purification kit, Himedia) and tested for influenza panel [A, B, A/H1N1pdm(2009) and A/H3] and RSV using the TRUPCR Flu panel with RSV kit, 3B Blackbio, India. SARS-CoV-2 was tested using TRUPCR SARS-CoV-2 kit, 3B Blackbio, India. A confirmed case is defined as a subject meeting the case definition with laboratory-confirmed test, based on the kit's cycle threshold (Ct) cut-off. The Ct cut-off for influenza, RSV and SARS-CoV-2 were 40, 40, and 35, respectively.

A representative set of samples were tested for a respiratory pathogens panel (TRUPCR Respiratory Pathogen Panel Kit, 3B Blackbio, India) (34). The kit allows qualitative detection of 14 bacteria and 17 respiratory viruses. The bacteria include *Staphylococcus aureus*, *Streptococcus pneumoniae*, *Klebsiella pneumoniae, Mycoplasma pneumoniae*, *Streptococcus agalactiae*, *Streptococcus pyogenes*, *Acinetobacter baumannii*, *Pseudomonas aeruginosa*, *Legionella pneumophila*, *Salmonella spp*, *Bordetella spp*, *Chlamydia pneumoniae*, *Haemophilus influenzae (A-F)* and *Moraxella catarrhalis*. The viruses include human adenovirus, human bocavirus, human coronavirus (alpha & beta), enterovirus, influenza A, influenza B, influenza A(H3N2), influenza A/H1N1pdm2009, human parainfluenza virus 1, human parainfluenza virus 2, human parainfluenza virus 3, human parainfluenza virus 4, human parechovirus and human RSV (A/B). RNA/DNA was extracted using Total Viral Nucleic Acid Extraction kit, 3B Blackbio. Amplification was performed on CFX96 Real-time PCR detection system (Biorad, USA) following the manufacturer's instructions. The VRDL send the cycle threshold values and the interpretation electronically to the investigator at Indian Institute of Technology Kharagpur.

### 2.5 Data management and analysis

All patient data was maintained on CDC Epi info^TM^ by a trained staff. Assuming a 2% influenza prevalence, the 95% CI lies between 3.2% and 0.8%; or 1.8% and 0.2% if the prevalence is 1%. Only investigator and the study staff involved in data management had access to patient data and laboratory results. Periodically data was exported into external hard drives. In each paediatric clinic, we recorded the total number of all outpatient department (OPD) patient visits, the number of patients who met the WHO ILI case definition and the number of patients enrolled. Through the hospital information system, we recorded the weekly total number of patients presenting to the OPD.

Seasons were categorized according to the Indian Meteorological Department into summer (March–May), monsoon (June–September), autumn/post-monsoon (October–November) and winter (December–February) (35). District West Midnapore faces a humid tropical monsoon climate where temperatures vary from an average of 19.3°C in winters to 32.2°C in summers. The district has a high relative humidity 59.17% (average), and an average annual rainfall of 1,485 mm (36). The socioeconomic status was measured by the Kuppuswamy Socioeconomic Scale 2021 which included data on education and occupation of the head of family and monthly family income (37). During analysis, family income and socioeconomic scale were recoded from lowest to highest. Other socioeconomic variables included the highest educational qualification of mother, mother's occupation, type of fuel used for cooking to understand pollution exposure, total number of people in house to understand overcrowding, and the total number of children in house. The geographic location of study participants was plotted using Quantum GIS software.

Categorical variables are presented as frequencies (%) and continuous variables as median (interquartile range, IQR). Comparisons were made using chi square (chi2) test for categorical variables. To examine the risk factors for hospitalization or association between pathogens, logistic regression was used to estimate the unadjusted odds ratios (OR) and 95% confidence intervals (CI). Statistical significance was defined as *p* < 0.05. Statistical analyses were performed using STATA/BE 17.0 (STATA Corp, Texas, USA).

### 2.6 Ethics approval

This study involves human participants and was approved by the Institute Ethical Committee of Indian Institute of Technology Kharagpur (#IIT/SRIC/DeanSRIC/2021) and the Institutional Ethics Committee of Midnapore Medical College (#IEC/2021/02). Approvals were obtained from medical superintendents of the three hospitals, and the district (#1655) and state health department [554#SS(ME)/Spl./184/2021].

## 3 Results

### 3.1 Proportion of ILI in outpatient clinics

Year-round ILI activity was observed among children of all ages. The online Supplementary Figure S1 shows the weekly ILI activity among children 0–14 years visiting MMCH and KSDH from April 1, 2022 to March 31, 2023. The frequency of ILI cases increased from a median of 3 cases per visit from April–July, to 10 in August, 11 in October, and 17 in November. From January 2023 onwards, ILI presentations increased from 10 in January, to 14 in February-March. The online Supplementary Figure S2 shows the total number of ILI cases by age group across one year. Across the year, ILI cases were observed more in the 0–2-year age group as compared to the older age groups.

In the one-year surveillance, with 91 sentinel site visits (65 to MMCH and 26 to KSDH), a total of 9,923 children under 15 years visited the paediatric clinics. Of 9,923, 1,001 fulfilled the ILI case definition. Of 1,001, the majority were found in 0–2-year age group 439 (44%), followed by >2-5-year age group 288 (29%), and >5–14-year age group 274 (27%), *p* = <0.001. Table 1 describes the cumulative proportion of ILI age-wise as 439/4,310 [10.2% (95% CI: 9.29–11.1)] in the 0–2-year-olds, 288/2,473 [11.6% (95% CI: 10.4–12.9)] in >2-5-year-olds, and, 274/3,140 [8.7% (95% CI: 7.7–9.7)] in >5–14-year-olds. Population level incidence of ILI could not be calculated due to non-availability of data on the total population at risk during the time period.

**Table 1:** Proportion of ILI in children attending the paediatric outpatient clinics of midnapore medical college & hospital (MMCH) and Kharagpur Sub divisional hospital (KSDH) in district west Midnapore from April 1, 2022 to march 31, 2023.

Parameter	0–24 months	25–60 months	>60 months-14 year	All ages
Number of children that visited the two outpatient clinics for all illnesses	23,239	14,459	18,524	56,222
Number of children screened for ILI (A)	4,310	2,473	3,140	9,923
Number of children that met ILI case definition (B)	439	288	274	1,001
Proportion of children that met ILI case definition % (95% CI) (P) = (A)/(B) × 100	10.2 (9.29–11.1)	11.6 (10.4–12.9)	8.7 (7.7–9.7)	10.1 (9.5–10.7)

ILI, influenza-like illness.

### 3.2 Enrollment

Overall, three hundred ninety children (0–2 years) with ILI were enrolled (260 from MMCH, 130 from KSDH), representing nearly 89% of ILI in children under-2. Maximum enrollment occurred from October-November (*n* = 99). The majority came from West Midnapore and a few (*n* = 4) from the adjoining districts. Online Supplementary Figure S3 presents the geographic location of study participants.

### 3.3 Individual and family characteristics

The individual and sociodemographic characteristics of the enrolled participants are tabulated in online Supplementary Table S1. Of 390, 76 (19%) were aged 0–6 months, 147 (37.6%) were 7–12 months and 167 (43%) were 13–24 months of age. There was a slight male predominance (217:173).

They came from families having a median of 5 members (95% CI: 4, 6). A few (14/390, 3.6%) families had tuberculosis at home. Less than half (48.6%) used both liquefied petroleum gas (LPG) and wood as a fuel for cooking, suggesting exposure to indoor air pollution. Others used LPG (32%), wood (18%), and, a few used wood and animal dung cakes (1.5%).

Around 7.87% of mothers and 5.74% of fathers were illiterate. The median number of years of school education of mothers was greater than the fathers [10 (8, 11) vs. 4 (3, 5)]. Most of the fathers were skilled agriculture and fishery workers. The majority came from upper-lower socioeconomic households having a median monthly family income of INR 12,560.

### 3.4 Medical and vaccination history of children and their mothers

More than one-third of children were born via caesarean (35%), a few were premature (4.8%) (online Supplementary Table S1). Nearly 24.7% were born low birth weight. Most of them (297/390) did not have any underlying condition. One had HIV and two had epileptic seizures. The majority (97.7%) were vaccinated through the universal immunization program (UIP). Vaccination record of individual vaccines were not recorded. A few (4/390, 1%) had received vaccines by paying out of pocket, outside of the UIP. Most of the mothers 341/388 (88%) had received COVID-19 vaccines during pregnancy. None had received influenza or hepatitis B vaccine, while 1/388 had received pertussis vaccine.

### 3.5 Illness details

Participants had been sick for a median of 2 days (95% CI: 2, 3) and presented with a median of 5 symptoms (IQR: 4-7) (online Supplementary Table S2). Fever, rhinorrhea, cough, nasal congestion and sneezing were most commonly reported: 98%, 85.6%, 84.6%, 69% and 43%, respectively. As compared to children >6 months of age, children 0-6 months of age presented more with sneezing (52.6% vs. 41.7%, *p* = 0.085) and wheezing (35.6% vs. 26.1%, *p* = 0.10). Their median body temperatures were 98.3°F (IQR: 97.9–98.8). Few (*n* = 7) needed nebulization. Antibiotic therapy was commonly initiated (79%), including amoxycillin-clavulanic acid (penicillins), azithromycin (macrolides) and cefuroxime/cefpodoxime/cefiximes (cephalosporins).

Some children (17%) had a family member sick with similar illness. Of the sick, the majority were mothers (37%) and siblings (36%).

### 3.6 Laboratory findings

Around 60% (234/390) of the samples had no pathogen. More than one-fourth (104/390) had a single pathogen, while 52/390 (13.3%) had multiple pathogens. Viruses were detected in 91/390 (23.3%), occurring singly (15%) or with other viruses (1.3%). RSVA/B was the major viral cause 49/390 (12.6%), followed by influenza 26/390 (6.6%), and SARS-CoV-2 3/390 (0.77%) (Table 2). The influenza subtypes included IA/H3 (13/26), IA/H1N1pdm2009 (9/26) and IB (4/26). Seven RSV positive samples were subtyped, of which six were RSVB and one RSVA. Influenza and RSV were co-detected in two.

**Table 2:** Proportion of respiratory pathogens in the nasopharynx of children with ILI age wise.

Pathogen	Overall (%)	1–6 month	7–12 month	13–24 month	*p* value*	*p* value*	*p* value*
		A	B	C	Between A & B	Between A & C	Between B & C
*N*	390	76	147	167			
Total Influenza	26 (6.6)	2 (2.6)	11 (7.5)	13 (7.8)	0.14	0.12	0.92
Influenza singly	20 (5.1)	2 (2.6)	9 (6)	9 (5.4)	0.25	0.33	0.78
Total Respiratory Syncytial Virus A/B	49 (12.6)	9 (11.8)	19 (13)	21 (12.6)	0.81	0.87	0.92
RSV singly	40 (10.2)	8 (10.5)	15 (10.2)	17 (10.2)	0.94	0.93	0.99
SARS-CoV-2	3 (0.77)	1 (1.3)	1 (0.7)	1 (0.6)	0.63	0.56	0.92
Influenza subtypes	*N* = 26						
IA/H3	13 (50)	2 (2.6)	4 (2.7)	7 (4.2)	0.97	0.55	0.48
IA/H1N1pdm	9 (34.6)	0	5 (3.4)	4 (2.4)	NA	NA	0.59
IB	4 (15.4)	0	2 (1.4)	2 (1.2)	NA	NA	0.89
Influenza subtype or RSV co-infection							
IA/H3 + IA/H1N1pdm + IB	2 (0.5)	0	0	2 (1.2)	NA	NA	NA
IA/H3 + IB	1 (0.2)	0	1 (0.7)	0	NA	NA	NA
IA/H1N1pdm + RSV	1 (0.2)	0	1 (0.7)	0	NA	NA	NA
IA/H3 + IB + RSV	1 (0.2)	0	1 (0.7)	0	NA	NA	NA
Other pathogens							
*N*	135	30	60	45			
* Streptococcus pneumoniae*	75 (55.5)	19 (63)	31 (52)	25 (55.5)	0.3	0.5	0.7
* Hemophilus influenzae*	39 (29)	7 (23)	19 (32)	13 (28.8)	0.41	0.6	0.76
* Moraxella catarrhalis*	5 (3.7)	2 (6.6)	1 (1.6)	2 (4.4)	0.21	0.67	0.4
* Staphylococcus aureus*	2 (1.5)	1 (3.3)	0	1 (2.2)	NA	0.77	NA
* Bordetella spp*	2 (1.5)	1 (3.3)	1 (1.6)	0	0.61	NA	NA
* Pseudomonas aeruginosa*	2 (1.5)	1 (3.3)	1 (1.6)	0	0.61	NA	NA
* *Parainfluenza virus 1	3 (2.2)	1 (3.3)	0	2 (4.4)	NA	0.8	NA
* *Parainfluenza virus 2	2 (1.5)	0	0	2 (4.4)	NA	NA	NA
* *Parainfluenza virus 3	6 (4.4)	1 (3.3)	5 (8.3)	0	0.37	NA	NA
* *Metapneumovirus	2 (1.5)	0	2 (3.3)	0	NA	NA	NA
* *Enterovirus	2 (1.5)	1 (3.3)	0	1 (2.2)	NA	0.77	NA
* *Rhinovirus	2 (1.5)	0	0	2 (4.4)	NA	NA	NA
* *Bocavirus	5 (3.7)	1 (3.3)	3 (5)	1 (2.2)	0.72	0.77	0.46
* *Adenovirus	4 (3)	0	2 (3.3)	2 (4.4)	NA	NA	0.77

RSV, respiratory syncytial virus; SARS-CoV-2, severe acute respiratory syndrome coronavirus 2; IA/H3, influenza A subtype H3; IA/H1N1pdm, influenza A subtype H1N1 pandemic 2009; IB, influenza B type.

* *p* values were calculated by the chi2 test.

**Figure 1:**
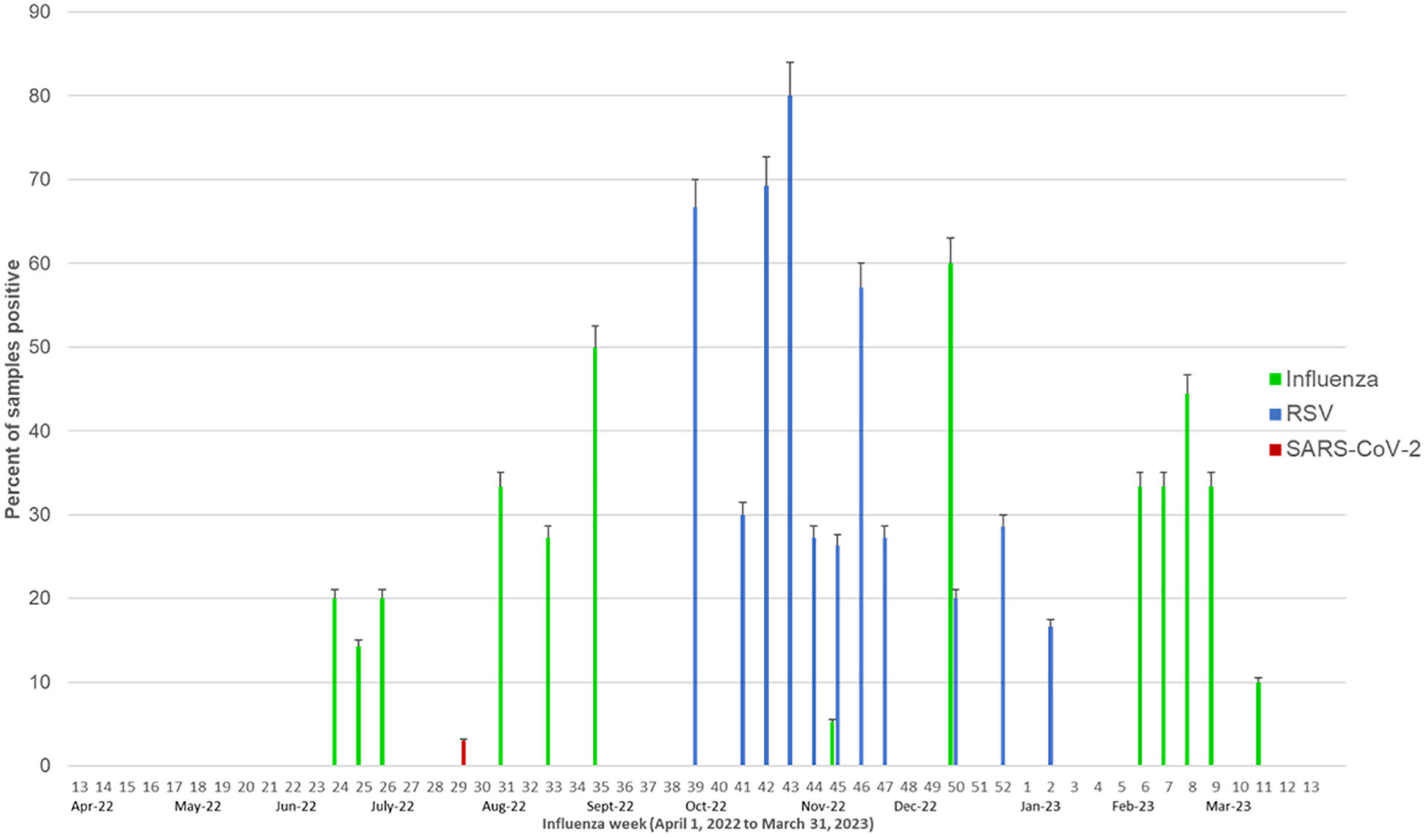
Weekly influenza, RSV and SARS-CoV-2 activity. The green bars represent the percent of samples positive for influenza, blue bars represent the percent of samples positive for RSV and red bars represent the percent positive for SARS-CoV-2.

Figure 1 depicts the weekly activity of influenza, RSV and SARS-CoV-2. The weekly mean flu positivity rate was 7.5% (0–2.6), which increased during epi weeks 24–35 (June–Sept 2022) and 6–9 (February 2023). The weekly average RSV positivity rate was 8.8% (0–0), which increased during epi weeks 39–47 (October-November 2022).

A representative sample set (135/390) was tested for the respiratory panel. Samples were randomly selected from each week. The majority of samples were from summer-22, monsoon and autumn season (90.3%) and remaining from winter (9.6%). Due to fund constraints, we could not test samples of March 2023. *Streptococcus pneumoniae* was the most common bacteria (75/135, 55.5%) followed by *Haemophilus influenzae* (39/135, 29%), *Moraxella catarrhalis* (5/135, 3.7%), *Staphylococcus aureus* (2/135, 1.5%), *Bordetella* spp (2/135, 1.5%) and *Pseudomonas aeruginosa* (2/135, 1.5%). The other respiratory viruses encompassed parainfluenza virus 3 (6/135, 4.4%); parainfluenza virus 1 (3/135, 2.2%); parainfluenza virus 2 (2/135, 1.5%), adenovirus (4/135, 3%), bocavirus (5/135, 3.7%), rhinovirus (2/135, 1.5%), metapneumovirus (2/135, 1.5%) and enterovirus (2/135, 1.5%). The proportion of virus and bacteria were not statistically different in between the 0–6 months, 7–12 months and >1 year age groups.

### 3.7 Clinical symptoms by viral cause

Children aged 0–6 months with RSV and influenza presented with more respiratory symptoms. Although the differences were not statistically significant, we noted children aged 0–6 months having RSV displayed more symptoms of sneezing, nasal congestion, rhinorrhea, wheezing, difficulty breathing and cough, as compared to the children >6 months of age (78%, 100%, 100%, 33%, 44%, 100%, vs. 45%, 70%, 82.5%, 25%, 40%, 97.5%, respectively). Similar trends were seen during influenza infection for difficulty breathing, sneezing, wheezing and cough (50%, 100%, 50%, 100% vs. 8%, 50%, 21%, 92%, respectively).

### 3.8 Co-infections

Single and co-infections of bacteria and viruses are listed in Table 3. Bacteria were detected in 92/135 (68%) cases, singly (33.3%), or co-detected with other bacteria (14.8%) or viruses (20%), mostly RSV, parainfluenza virus 3 and influenza virus. *S. pneumoniae* was most common co-detection (40/135, 29.6%) followed by *H. influenzae* (32/135, 23.7%), both of which were co-detected together (*n* = 13), or with other bacteria (*n* = 3) or viruses (*n* = 9).

**Table 3:** Single and co-infections in the nasopharynx of children with ILI while studying the respiratory pathogens profile (*N* = 135).

Pathogen	*n* (%)
*N*	135
No pathogen	**36** (**26.6%)**
One bacterium, *N* = 135	**45** (**33.3%)**
One pathogen, *N* = 135	51 (37.7%)
*Streptococcus pneumoniae*	35 (26%)
*Hemophilus influenzae*	7 (5.2%)
Metapneumovirus	2 (1.5%)
*Staphylococcus aureus*	1 (0.7%)
*Pseudomonas aeruginosa*	1 (0.7%)
*Moraxella catarrhalis*	1 (0.7%)
Parainfluenza virus 1	1 (0.7%)
Parainfluenza virus 2	1 (0.7%)
Parainfluenza virus 3	1 (0.7%)
Bocavirus	1 (0.7%)
More than one pathogen, *N* = 135	47 (35%)
Bacteria + Bacteria, *N* = 47	20 (42.5%)
*S. pneumoniae* + *H. influenzae*	13 (27.6%)
*S. pneumoniae* + *Bordetella spp*	2 (4.2%)
*S. pneumoniae* + *M. catarrhalis*	2 (4.2%)
*S. pneumoniae* + *H. influenza*e + *M. catarrhalis*	2 (4.2%)
*S. pneumoniae* + *H. influenzae* + *P. aeruginosa*	1 (2%)
1 Bacterium + ≥ 1 Virus, *N* = 47	17 (36%)
*H. influenzae* + RSV	4 (8.5%)
*S. pneumoniae* + RSV	2 (4.2%)
*S. pneumoniae* + Bocavirus	2 (4.2%)
*S. pneumoniae* + Parainfluenza virus 1	2 (4.2%)
*S. pneumoniae* + Parainfluenza virus 3	1 (2%)
*H. influenzae* + Adenovirus	1 (2%)
*H. influenzae* + Influenza A/H3N2	1 (2%)
*H. influenzae* + Parainfluenza virus 3	1 (2%)
*S. pneumoniae* + Enterovirus	1 (2%)
*S. pneumoniae* + Influenza A/H1N1pdm09	1 (2%)
*S. pneumoniae* + RSV + Adenovirus + Bocavirus	1 (2%)
> 1 Bacteria + ≥ 1 Virus, *N* = 47	10 (21.3%)
*S. pneumoniae* + *H. influenzae* + Rhinovirus	2 (4.2%)
*S. pneumoniae* + *H. influenzae* + Parainfluenza virus 3	2 (4.2%)
*S. pneumoniae* + *S. aureus* + Parainfluenza virus 3	1 (2%)
*S. pneumoniae* + *H. influenzae* + Adenovirus	1 (2%)
*S. pneumoniae* + *H. influenzae* + Influenza A/H1N1pdm	1 (2%)
*S. pneumoniae* + *H. influenzae* + Influenza A/H1N1pdm + Bocavirus + Adenovirus	1 (2%)
*S. pneumoniae* + *H. influenzae* + Parainfluenza virus 2	1 (2%)
*S. pneumoniae* + *H. influenzae* + RSV	1 (2%)

*S. pneumoniae, Streptococcus pneumoniae; H. influenzae, Haemophilus influenzae; M. catarrhalis, Moraxella catarrhalis; P. aeruginosa, Pseudomonas aeruginosa*; RSV, Respiratory Syncytial Virus; Influenza A/H3N2, Influenza A subtype H3N2; Influenza A/H1N1pdm, Influenza A subtype H1N1 pandemic 2009.

RSV was co-detected with *H. influenzae* (*n* = 4), *S. pneumoniae* (*n* = 2), *S. pneumoniae* + bocavirus + adenovirus (*n* = 1) and *S. pneumoniae* + *H. influenzae* (*n* = 1). Influenza virus was co-detected with *S. pneumoniae* (*n* = 1), *H. influenzae* (*n* = 1), *S. pneumoniae* and RSV (*n* = 1), *S. pneumoniae* and *H. influenzae* (*n* = 1) and *S. pneumoniae* + *H. influenzae* + Bocavirus + Adenovirus (*n* = 1). SARS-CoV-2 was detected singly. Eight children needed nebulization of which two had RSV, one had *S. pneumoniae* + *H. influenzae* + RSV and one had *H. influenzae*.

A child living with HIV was positive for *S. pneumoniae*, *H. influenzae* and adenovirus. Children coming from tuberculosis households tested positive for multiple pathogens viz., RSV (*n* = 2), *S. pneumoniae* (*n* = 2)*,* influenza A H1N1pdm (*n* = 1), SARS-CoV-2 (*n* = 1), parainfluenza virus 1 (*n* = 1), *S. pneumoniae* and *H. influenzae* (*n* = 1)*,* and, *S. pneumoniae* and *Bordetella* spp (*n* = 1).

### 3.9 Seasonality of viruses

**Figure 2:**
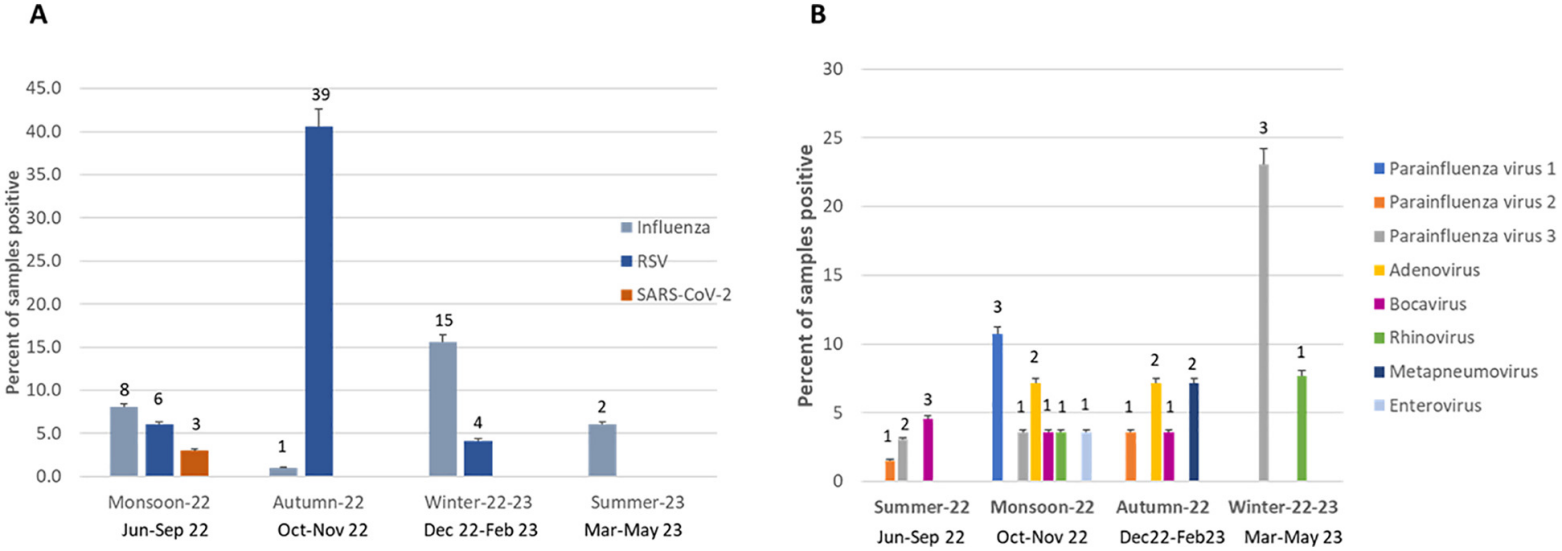
Seasonality of respiratory viruses **(A)** seasonality of influenza, RSV and SARS-CoV-2 in children (*n* = 390) **(B)** seasonality of other respiratory viruses (*n* = 135).

We sampled equivalent number of samples across all seasons (24%-25%, each). Influenza activity increased during monsoon and winter (Figure 2A). Influenza A H1N1/pdm09 predominated (6/9) in the 2022 monsoon while Influenza A H3 (9/13) and Influenza B (4/4) in the 2023 winter. RSV mainly peaked (39/49) during autumn (October–November). SARS-CoV-2 was observed only in monsoon, coinciding with India's July 2022 omicron sub-variant (BA.2.75) wave. Low levels of other viruses were detected in the monsoon (see Figure 2B) with parainfluenza virus 3 found in winter, monsoon and summer; parainfluenza virus 1 in monsoon only; adenovirus in monsoon and autumn; and metapneumovirus in autumn.

### 3.10 Patient disposition

The majority of children (95.6%) were discharged home. Seventeen (4.4%) required hospitalization, of which five were 0-6 months old, seven were 7–12-month-old, and, five were >13-month-old. Most (11/17) hospitalized children belonged to the upper-lower socioeconomic status. RSV was the most common (5/17) pathogen, including two in infants aged 0-6 months with low birth weights. None had influenza or SARS-CoV-2.

Of the 17, seven were tested for respiratory panel. Findings included: parainfluenza virus 3 (*n* = 1), *H. influenzae (n* *=* *1)*, *S. pneumoniae (n* *=* *2)*, RSV and *H. influenzae (n* *=* *1)*, *S. aureus* and *S. pneumoniae* and parainfluenza 3 virus (*n* = 1) and *S. pneumoniae* and *H. influenzae* and RSV (*n* = 1).

### 3.11 Risk factors for hospitalization

Difficulty breathing was associated with a four-fold increased risk of hospitalization (OR = 4.47, 95% CI: 1.67–12), see online Supplementary Table S3. Children positive for RSV (OR = 3.11; 95% CI: 1–9.26), *S. aureus* (OR = 21.2, 95% CI: 1.17–381) and parainfluenza virus 3 (OR = 12.4, 95% CI: 1.8–84.4) had increased odds of hospitalization. Trends toward increased hospitalization were also observed in children experiencing a greater number of symptoms (OR = 1.27, 95% CI: 0.98–1.65, *p* = 0.06) and with viral detection (OR = 2.4, 95% CI: 0.89–6.5, *p* = 0.08).

### 3.12 Risk factors for respiratory pathogens associated with ILI

Cough was found an indicator of viral infections (OR = 3.2, 95% CI: 1.23–8.3, *p* = 0.017) and nasal congestion for bacterial (OR = 2.7, 95% CI: 1.5–5, *p* = 0.001) and viral-bacterial detections (OR = 12.36, 95% CI: 1.6–92, *p* = 0.014). Risk factors for RSV, influenza and SARS-CoV-2 infections are summarized in online Supplementary Table S4. Autumn season increased the risk of RSV (OR = 19.4, 95% CI: 9.1–41.16, *p* = <0.001). Symptoms of cough and difficulty breathing increased the risk of RSV 8.4-fold and 2.98-fold, respectively. Higher family income was associated with lower RSV rates (OR = 0.44, 95% CI: 0.24–0.82). The likelihood of RSV infections increased with viral-bacterial co-detections, 3.3 times.

On similar lines, influenza infections increased during winter, multiple co-detections and viral-bacterial co-detections by 2.6-fold, 6.8-fold and 3.3-fold, respectively. Being in tuberculosis households increased the risk of SARS-CoV-2 in children (OR = 14.3, 95% CI: 1.2–168, *p* = 0.03). Interestingly, SARS-CoV-2 increased with increase in household income (OR = 7.9, 95% CI: 1.3–67, *p* = 0.02). Rhinorrhea was less common in children with SARS-CoV-2 (OR = 0.07, 95% CI: 0.006–0.85, *p* = 0.03), while decreased urine output was more (OR = 21, 95% CI:1.7–253, *p* = 0.017).

Nasal congestion was associated with *S. pneumoniae* detection (OR = 2.7, 95% CI:1.2–6, *p* = 0.012). Wheezing symptom increased the risk of parainfluenza virus 3 infection (OR = 7, 95% CI:1.31–38, *p* = 0.02) (online Supplementary Table S5). Parainfluenza virus 3 and *S. aureus* exhibited positive association (OR = 25.6, 95% CI:1.4–471, *p* = 0.03). Difficulty breathing, nasal congestion and rhinorrhea increased the risk of *H. influenzae* 5.6-fold, 6.2-fold and 8.17-fold, respectively. Similar illness within the family increased the risk of finding *H. influenzae*, 7-fold.

## 4 Discussion

This study examines the incidence of ILI, the prevalence of influenza, RSV, and SARS-CoV-2, and the other etiologies of ILI among children 0–2 years of age. The study was conducted when India had experienced three major COVID-19 waves (Wuhan, Delta, Omicron), COVID-19 vaccines were widely administered, including to pregnant women, and the non-pharmaceutical measures had been lifted. This period was chosen to study the dynamics of influenza and RSV activity in the post-pandemic phase. Highest level of ILI was observed in children 0-2 years of age group as compared to the >2-5 year and >5–14-year age groups. RSV emerged as the most common viral pathogen, followed by influenza, and, SARS-CoV-2 was low.

While most previous ILI research in West Bengal focused on urban Kolkata, to our knowledge this study is the first from the rural area of West Midnapore, approximately 130 km west of Kolkata. Similar to our study, a pan-India surveillance of influenza and SARS-CoV-2 from July 2021 to October 2022 reported influenza prevalence of 5.9% in infants and 12% in children aged 1–5, with low SARS-CoV-2 positivity (3.8% in infants and 2% in 1–5-year-olds) (38). Pre-pandemic studies from northern India in 2011 demonstrated that influenza accounted for 7.6% (95% CI: 4.4–12) of ILI among 0–4-year-olds visiting clinics (39).

Our study observed influenza peaks during the monsoon and winters, aligning with prior studies showing similar activity (July-September) in Bengal and northern India (17, 39). A/H1N1pdm09 predominated in the 2022 monsoon season in this study, in line with pan-India ILI and severe acute respiratory illness (SARI) studies (38) and a central India study indicating H1N1pdm09 (20.9%) dominance in adults from July-December 2022 (40). Conversely, a study from southern India between June-August 2022 found influenza B (76.5%) and SARS-CoV-2 (12.2%) prevalence in children (41). From January to March 2023, a major A/H3N2 wave was observed in India, aligning with our findings (42).

A recent study from Italy identified cough as a predictor of viral infections, concurring with our findings (43). Our study highlights the high prevalence of RSV, which aligns with the global post-pandemic RSV surge during 2022, and the burden it imposes on paediatric hospital settings (10, 23, 24). In Ethiopia, a 16.2% RSV positivity rate was observed in children under-5 during 2021-2022 (44). However, a study from Western India reported lower RSV positivity rates: 5.5% in 2022 and 6.2% in 2023, though the 0–4-year age group remained the most affected (27). We found children having cough and difficulty breathing were at increased risk for RSV and 29% of those hospitalized had RSV. In concurrence, a study from Egypt reported RSV associated with higher rates of dyspnea in children under-2 and under-5 as compared to >5 year age groups (23). In the US, RSV was associated with a 70% increased need of advanced respiratory support among children during 2022–2023, as compared to the pre-pandemic season (45). A pneumonia etiology research study from Gambia among hospitalized children found RSV (37%), as the primary cause of severe pneumonia, and pneumococcus as more common in the most severe cases (46). These findings highlight the more severe disease caused by RSV than influenza among young children and its burden on the health system. Overall, this also underscores the importance of integrating RSV surveillance within the routine influenza and SARS-CoV-2 surveillance, in accordance with the WHO guidelines (47).

Early in the COVID-19 pandemic, SARS-COV-2 was considered to cause a mild disease in children (48). However, the emergence of omicron variant revealed increased transmissibility and severity among young children (49). We detected low levels of SARS-CoV-2 among children, only during the surge of the omicron subvariant (BA.2.75) in India. This could be due to the high (88%) maternal COVID-19 vaccination coverage.

The pneumococcal carriage rates in our study were similar to a study from Laos (55.4%) among children under-5 through a community-based ILI surveillance study (50). Our study revealed multiple co-detection of bacteria and viruses, which did not correlate with symptom severity or hospitalization, as seen elsewhere (51). Similar to our observations, previous studies demonstrate *S. pneumoniae* and *H. influenzae* as common co-infecting pathogens in ARIs (52, 53). Viral infections from influenza, RSV and other viruses can predispose individuals to secondary bacterial co-infections, leading to a more severe clinical course with increased morbidity and mortality (54). However, as our study, lacked specimens from asymptomatic children, or lower respiratory tract sources, we consider pneumococcal detection as a carriage rather than an infection or acquisition, knowing that bacteria usually colonize in asymptomatic children. Interestingly, we observed nasal congestion increased the risk of pneumococcus, and in a few hospitalized cases pneumococcus was detected. The detection of *H. influenzae,* might indicate an infection as we noted difficulty breathing was associated with *H. influenzae.* Furthermore, *H. influenzae* was found in those hospitalized singly in one case, with RSV in another case and with RSV and *S. pneumoniae* in the third case. This finding is interesting as the majority of children were vaccinated with vaccines covered in the Universal Immunization Program (UIP), including HiB vaccine. Future research on the types of *H. influenzae* circulating is needed.

The findings of this study are important for informing immunization policies. The implementation of maternal RSV vaccines (Pfizer) and long-acting RSV-specific monoclonal antibodies in high-income countries shows promise in preventing severe RSV-LRTI in young children (55–59). Maternal influenza immunization has been adopted in many countries demonstrating its protective effect on infants under six months (60, 61). Despite the burden, access to maternal influenza and RSV immunization, and, paediatric influenza immunization remains limited in LMICs, including India (62, 63). In India, maternal influenza immunization has been recommended by the Health Ministry and influenza immunization in children is considered “desirable”. However, both are not yet part of the UIP (63). Our findings emphasize the critical need for equitable access to these interventions.

The strength of this study is its setting in two outpatient clinics across two hospitals, screening approximately ten-thousand children, enrolling a large cohort of young children, and providing a robust view of the epidemiology in this underrepresented region. The inclusion of influenza, RSV and SARS-CoV-2 in ILI surveillance, is important due to their increased risk in children. This approach is in line with the global efforts of integrated respiratory virus surveillance. Using a respiratory panel provides a comprehensive understanding about the other ILI etiologies and co-infections in the paediatric population. The study followed a rigorous protocol with standardized procedures and maintained intense surveillance for one year to capture virus seasonality.

The study has some limitations. Lack of a hospital utilization survey and reliable population estimates prevented an assessment of the burden of influenza, RSV and SARS-CoV-2. We cannot draw strong conclusions about the clinical significance of bacterial nasopharyngeal carriage and viral-bacterial co-detections. Further, absence of data on the serotypes and density of pneumococcus and *H. influenzae* limits their association with clinical severity. Lack of information about the study participant's pneumococcal, Hib (H. influenzae type B) and pertussis vaccination coverage hinders us to draw correlations between their nasopharyngeal carriage and vaccination status. The current WHO ILI case definition considers time period for inclusion “within 10 days of symptom onset” (64), while we followed the old definition of sampling “within 3 days”. This modification aimed to enhance influenza and RSV detection, which was evident by our findings. The study relied on children visiting the study hospitals, which may not give a true picture of ILI prevalence in the community and the corresponding etiologies. Lastly, we didn't do a follow-up of study participants for illness outcome.

## 5 Conclusions

This study makes a meaningful contribution to the current knowledge base. The study highlights that children aged 0–2-years bear the greatest burden of ILI in eastern India, with RSV and influenza as key viral pathogens. Delineating the etiology of respiratory infections in children is important for improving the management, guiding antiviral prescriptions, informing vaccination timings, policy and reducing antimicrobial resistance. The study contributes to the global evidence on RSV and influenza prevalence in the paediatric population. These findings are useful for the clinicians and policy makers for making RSV and influenza vaccination decisions. Future research is needed on RSV epidemiology, subtypes and genotypes, paving the way for the development of vaccines and mononclonal antibodies tailored to India's needs. The clinical significance of viral-bacterial co-detections warrants further research. Multiplex testing for severe LRTIs would be useful in delineating the etiologies thereby guiding treatment and antibiotic usage, although, its cost remains a constraint in LICs and LMICs. Our study noted the lack of maternal influenza immunization practice in the region. The presence of multiple pathogens among children from high-risk TB affected households demands careful interventions.

## Data Availability

The original contributions presented in the study are included in the article/Supplementary Material, further inquiries can be directed to the corresponding author.
