# Supplementary material for: Molecular epidemiology of influenza, respiratory syncytial virus, SARS-CoV-2, other respiratory viruses and bacteria among children 0–2-year-olds in West Bengal: a one-year influenza-like illness surveillance study (2022–2023)
Source: Front Epidemiol. 2025 May 20;5:1578951. doi: 10.3389/fepid.2025.1578951 (PMC12129922; doi:10.3389/fepid.2025.1578951)
Supplement: Supplementary file 1 [file Table1.docx]

**Supplementary Materials**

**
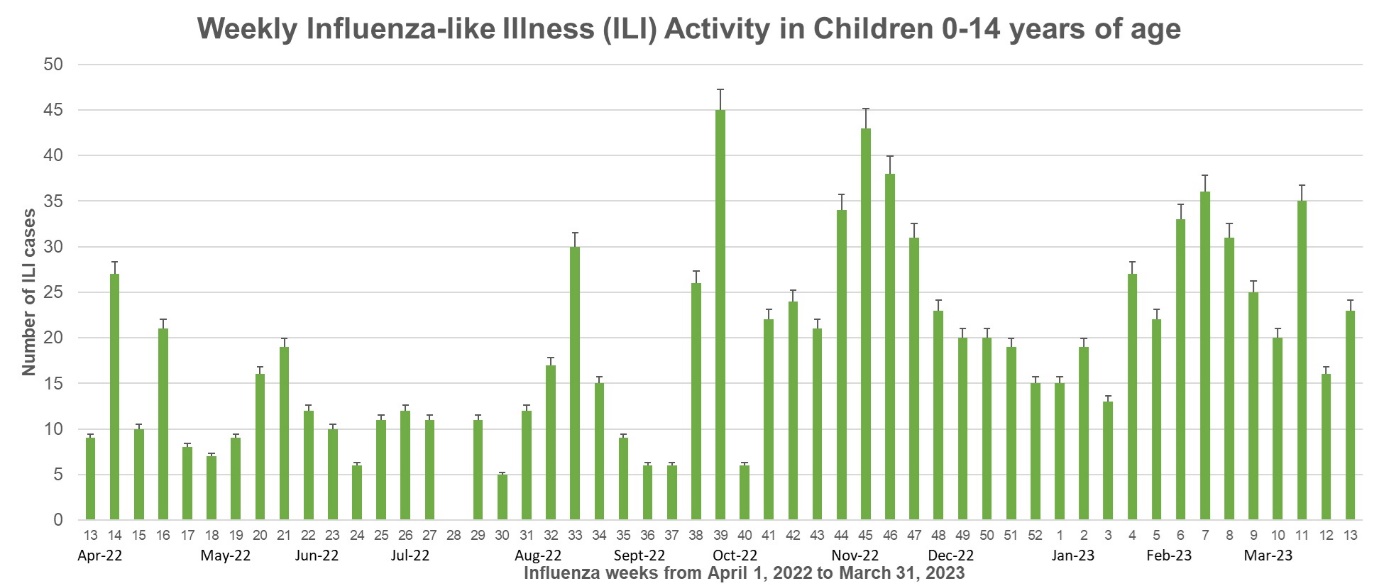
**

**Figure S1**: Weekly influenza-like illness (ILI) presentations of children 0-14 years of age in two sentinel sites from April 1, 2022 (epi week 13) to March 31, 2023 (epi week 13).

**
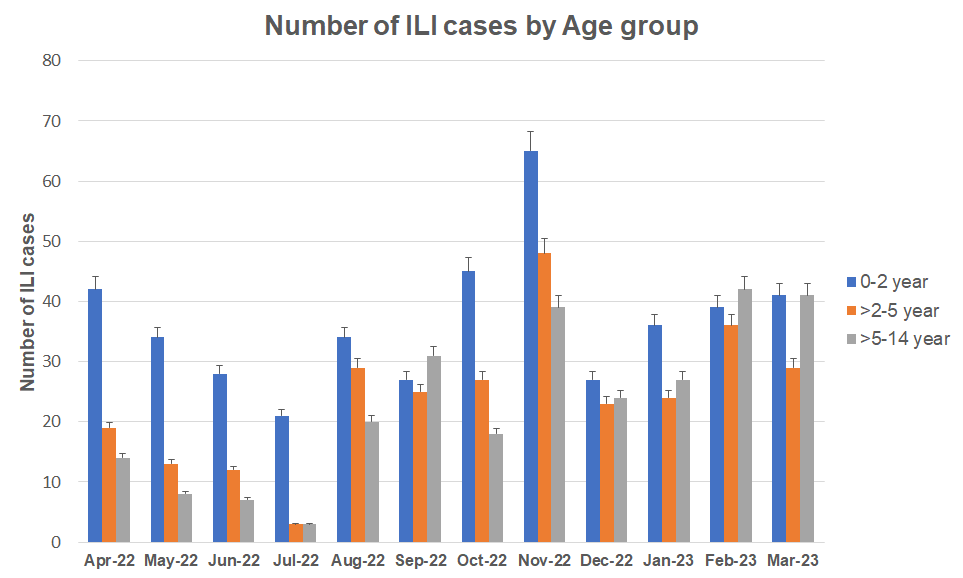
**

**Figure S2**: Monthly ILI presentations in children age-wise. The blue bars represent the total number of ILI cases in the 0–2-year age group, orange bars represent the total number of ILI cases in the >2-5-year age group and the grey bars represent the total number of ILI cases in the >5-14-year age group.


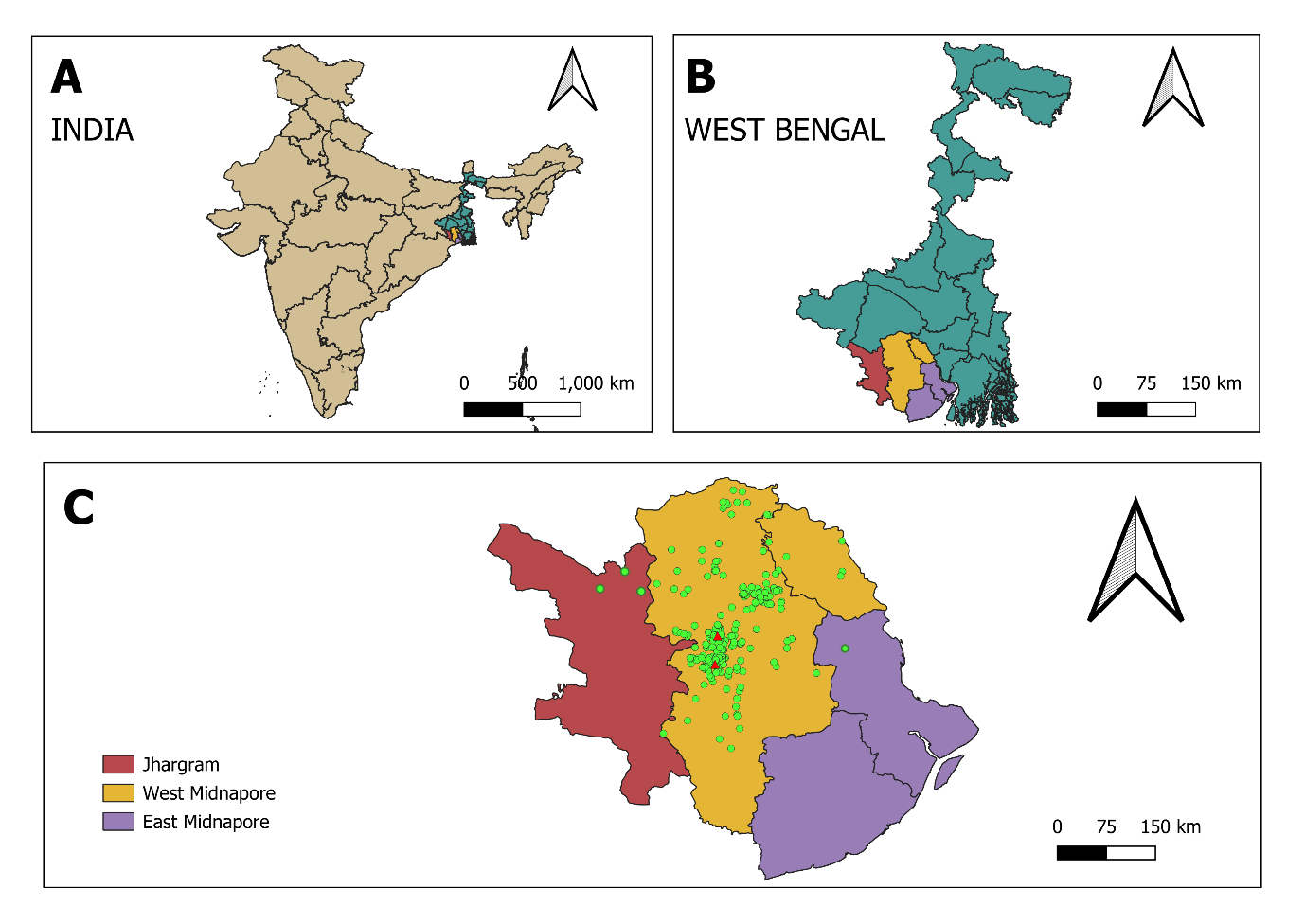


**Figure S3**: Geographic location of study participants (n=390). A. Political map of India B. Map of West Bengal C. Map of West Midnapore (yellow), East Midnapore (purple) and Jhargram (Rust). The green circles represent the location of residence of individual study participants and the red triangles are the Midnapore Medical College & Hospital and the Kharagpur Sub Divisional Hospital.

**Table S1**: Sociodemographic characteristics, birth history and maternal vaccination details of study participants

| **Characteristic** | **Detail** | **n (%) or median (IQR)** |
| --- | --- | --- |
| N |  | 390 |
| Enrollment, N=390 | KSDH | 130 (33.3%) |
|  | MMCH | 260 (66.6%) |
| Age in months, N=390 | Median (IQR) | 12 (7, 18) |
|  | 1-6 month | 76 (19%) |
|  | 7-12 month | 147 (37.6%) |
|  | 13-24 month | 167 (43%) |
| Gender, N=390 | Male | 217 (55.6%) |
|  | Female | 173 (44.4%) |
| Number of people at home, N=390 | Median (p25, p75) | 5 (4, 6) |
| Number of children within the household, N=389* | Median (p25, p75) | 1 (0, 1) |
| Birth history, | Premature birth, N=390 | 19 (4.8%) |
|  | Institutional delivery, N=389 | 371 (95.4%) |
|  | Caesarean delivery, N=389 | 137 (35%) |
|  | Birth weight, N=380 | 2.7 (2.5, 3) |
|  | Low birth weight, N=380 | 94 (24.7%) |
| Underlying conditions, N=390 | HIV | 1 (0.25%) |
|  | Seizures | 2 (0.5%) |
| Immunization through UIP, N=390 | Yes | 381 (97.7%) |
| Vaccination outside of UIP by out-of-pocket payment, N=390 | Yes | 4 (1%) |
| TB at home, N=390 | Yes | 14 (3.6%) |
| Fuel used for cooking, N=389 | LPG | 124 (32%) |
|  | Wood | 70 (18%) |
|  | LPG and wood | 189 (48.6%) |
|  | Wood and animal dung cakes | 6 (1.5%) |
| Mother’s education, N=381 | Median (p25, p75) | 10 (8, 11) |
| Mother’s occupation, N=381 | Home maker | 358 (94%) |
|  | Agriculture | 8 (2%) |
|  | Daily wager | 8 (2%) |
|  | Own Business | 2 (0.5%) |
|  | Tutor | 1 (0.26%) |
|  | Panchayat | 1 (0.26%) |
|  | Salaried job | 2 (0.5%) |
|  | Studying | 1 (0.26%) |
| Maternal vaccination history, N=388 | Influenza vaccine | 0 |
|  | COVID-19 vaccine | 341 (88%) |
|  | Pertussis vaccine | 1 (0.26%) |
|  | Hepatitis B vaccine | 0 |
| Father’s education, N=383 | Median grade (p25, p75) | Middle school (High school, Primary school) |
| Father’s occupation, N=383 | Median (p25, p75) | Agriculture (skilled worker, machine operator) |
| Family income, N=383 | Median (p25, p75), INR | 6175-18946 (6175-18496, 6175-18496) |
| Socioeconomic status**, N=383 | Median (p25, p75) | Upper lower (lower middle, upper lower) |

Abbreviations: IQR: Interquartile range; KSDH: Kharagpur Sub Divisional Hospital; MMCH: Midnapore Medical College & Hospital; HIV: Human Immunodeficiency Virus; UIP: Universal Immunization Program; TB: Tuberculosis; COVID-19: Coronavirus disease 2019; LPG: Liquified Petroleum Gas; INR: Indian rupees

*Some observations are missing as the children were orphan or adopted or the guardians were unwilling to answer; **Kuppuswamy Socioeconomic Status Scale 2021

**Table S2**: Clinical presentation of children with ILI symptoms

| **Clinical features** | n (n/N %) |
| --- | --- |
| N | 390 |
| **Symptoms on presentation** |  |
| Fever | 382 (98%) |
| Cough | 330 (84.6%) |
| Rhinorrhea | 334 (85.6%) |
| Nasal congestion | 269 (69%) |
| Sore throat | 4 (1%) |
| Difficulty breathing | 81 (21%) |
| Sneezing | 167 (43%) |
| Wheezing | 109 (28%) |
| Change in sleep pattern | 60 (15%) |
| Redness of eye | 42 (11%) |
| Loss of appetite | 153 (39%) |
| Decreased urine output | 11 (3%) |
| Change in activity | 14 (3.6%) |
| Vomiting/diarrhoea | 105 (27%) |
| Other | 3 (0.77%) |
| **Duration of sickness, days, median (IQR)** | 2 (2, 3) |
| **Similar sickness at home,** | 67 (17%) |
| Mother | 25 (37%) |
| Father | 4 (6%) |
| Sibling | 24 (36%) |
| Grandmother | 4 (6%) |
| Grandfather | 2 (3%) |
| Aunt | 4 (6%) |
| Uncle | 1 (1.5%) |
| Both parents | 1 (1.5%) |
| Grandmother and sibling | 1 (1.5%) |
| **Vitals** |  |
| Weight, kg, median (95% CI) | 8 (6.8, 9) |
| Temperature, °F | 98.3 (97.9, 98.8) |
| **Antibiotic prescribed** | 308 (79%) |

Abbreviations: *IQR: interquartile range, 95% CI: 95% Confidence Interval

**Table S3**: Risk factors for hospitalization

|  | **OR (95% CI)*** | **P value**** |
| --- | --- | --- |
| Age | 0.95 (0.87-1.03) | 0.21 |
| Gender | 0.67 (0.24-1.8) | 0.44 |
| Monsoon season | 0.38 (0.08-1.69) | 0.2 |
| Autumn season | 2.2 (0.82-6) | 0.113 |
| Winter season | 0.64 (0.18-2.3) | 0.5 |
| Sickness duration | 1.07(0.92-1.24) | 0.39 |
| Similar sickness at home | 1.03 (0.29-3.7) | 0.96 |
| Number of people at home | 1.00 (0.81-1.23) | 0.97 |
| Overcrowding (>6) at home | 0.92 (0.3-2.9) | 0.89 |
| Indoor air pollution | 1.3 (0.65-2.56) | 0.46 |
| Number of children within household | 1.27 (0.72-2.25) | 0.4 |
| Institutional delivery | 1.26 (0.4-4) | 0.69 |
| Premature birth | 2.84 (0.6-13.2) | 0.195 |
| Caesarean delivery | 1 (0.36-2.77) | 0.99 |
| Low birth weight | 0.39 (0.08-1.7) | 0.22 |
| Breastfeeding duration | 0.99 (0.92-1.07) | 0.92 |
| Family income | 0.7 (0.28-1.8) | 0.48 |
| Socioeconomic status | 0.8 (0.37-1.8) | 0.62 |
| Number of symptoms | 1.27 (0.98-1.65) | 0.06 |
| Rhinorrhea | 0.7 (0.2-2.6) | 0.62 |
| Nasal congestion | 2 (0.58-7.37) | 0.26 |
| Difficult breathing | **4.47 (1.67-12)** | **0.003** |
| Sneezing | 0.68 (0.25-1.9) | 0.47 |
| Wheezing | 0.54 (0.15-1.9) | 0.34 |
| Redness of eyes | 0.5 (0.06-4) | 0.51 |
| Vomiting/Diarrhoea | 2.36 (0.88-6.28) | 0.085 |
| Virus in the nasopharynx | 2.4 (0.89-6.5) | 0.08 |
| Bacteria in the nasopharynx | 1.82 (0.65-5) | 0.25 |
| Virus-bacteria co-detection in the nasopharynx | 3.1 (0.84-11.6) | 0.09 |
| *Staphylococcus aureus* | **21.2 (1.17-381)** | **0.038** |
| *Streptococcus pneumoniae* | 1.07 (0.23-5.00) | 0.93 |
| *Haemophilus influenzae* | 1.92 (0.4-9) | 0.41 |
| Parainfluenza virus 3 | **12.4 (1.8-84.4)** | **0.01** |
| Respiratory Syncytial Virus | **3.11 (1-9.26)** | **0.04** |

*OR (95% CI): Odds Ratio (95% Confidence Interval); **p values were calculated by bivariate logistic regression; values in bold indicate statistical significance

**Table S4**: Risk factors for nasopharyngeal detection of RSV, influenza and SARS-CoV-2 among children with ILI

|  | **Respiratory Syncytial Virus** | | **Influenza** | | **SARS-CoV-2** | |
| --- | --- | --- | --- | --- | --- | --- |
|  | **OR (95% CI)*** | **P value**** | **OR (95% CI)** | **P value** | **OR (95% CI)** | **P value** |
| Age | 0.99 (0.94-1.04) | 0.74 | 1.03 (0.96-1.1) | 0.39 | 0.92 (0.75-1.12) | 0.42 |
| Gender | 1.02 (0.56-1.8) | 0.93 | 0.66 (0.26-1.7) | 0.39 | 2.5 (0.23-28.1) | 0.45 |
| Monsoon season | 0.37 (0.15-0.9) | 0.03 | 2 (0.8-5.15) | 0.13 | NA |  |
| Autumn season | **19.4 (9.1-41.16)** | **<0.001** | 0.15 (.02-1.15) | 0.07 | NA |  |
| Winter season | 0.24 (0.08-0.68) | 0.008 | **2.66 (1.07-6.6)** | **0.036** | NA |  |
| Sickness duration | 1.04 (0.93-1.18) | 0.45 | 1.07 (0.94-1.23) | 0.28 | 1.1 (0.22-27) | 0.47 |
| Similar sickness at home | 1.09 (0.5-2.4) | 0.81 | 1.22 (0.4-3.7) | 0.73 | 2.4 (0.22-27) | 0.47 |
| Number of people at home | 1.02 (0.9-1.16) | 0.76 | 0.89 (0.7-1.13) | 0.34 | 0.62 (0.28-1.34) | 0.23 |
| Overcrowding (>6) at home | 1.24 (0.64-2.4) | 0.52 | 0.32 (0.07-1.4) | 0.133 | NA |  |
| Tuberculosis in family | 1.16 (.25-5.36) | 0.846 | 1.44 (0.18-11.6) | 0.73 | **14.3 (1.2-168)** | **0.03** |
| Indoor air pollution | 0.75 (0.5-1.14) | 0.18 | 0.75 (0.4-1.37) | 0.35 | 2.5 (0.47-13.7) | 0.28 |
| Number of children in the household | 0.79 (0.52-1.2) | 0.27 | 1.33 (0.8-2.2) | 0.28 | 1.57 (0.47-5.3) | 0.46 |
| Institutional delivery | 1 (0.48-2.11) | 0.97 | 2 (0.73-5.5) | 0.17 | 0.6 (0.03-10.77) | 0.73 |
| Caesarean delivery | 1.08 (0.58-2) | 0.81 | 1.9 (0.77-4.7) | 0.16 | NA |  |
| Premature birth | 1.93 (.61-6) | 0.26 | NA |  | NA |  |
| Birthweight | 0.87 (0.5-1.5) | 0.6 | 0.73 (0.3-1.826) | 0.5 | **0.52 (0.28-0.95)** | **0.03** |
| Low birth weight | 1.5 (0.77-2.9) | 0.22 | 1.56 (0.57-4.27) | 0.39 | 1.5 (0.1-17) | 0.73 |
| Breastfeeding duration | 0.97 (0.926-1.03) | 0.22 | 1 (0.96-1.1) | 0.48 | 0.94 (0.78-1.13) | 0.54 |
| Family income | **0.44 (0.24-0.82)** | **0.01** | 0.97 (0.42-2.2) | 0.94 | **7.9 (1.36-67.1)** | **0.02** |
| Socioeconomic status | 0.65 (0.4-1.08) | 0.09 | 1.04 (0.5-2.14) | 0.91 | 1.6 (0.26-9.6) | 0.61 |
| **Symptoms** |  |  |  |  |  |  |
| Cough | **8.4 (1.14-62.5)** | **0.037** | 1.4 (0.31-6.2) | 0.65 | NA |  |
| Rhinorrhea | 0.93 (0.4-2.2) | 0.88 | 1.44 (0.32-6.4) | 0.63 | **0.07 (0.006-8.5)** | **0.03** |
| Nasal congestion | 1.4 (0.7-2.77) | 0.35 | 1 (0.38-2.7) | 0.98 | 0.21 (0.02-2.4) | 0.21 |
| Sore throat | 2.3 (0.24-23) | 0.46 | 6.4 (0.64-64.8) | 0.11 | NA |  |
| Difficulty breathing | **2.98 (1.58-5.6)** | **0.001** | 0.63 (0.18-2.2) | 0.47 | NA |  |
| Sneezing | 1.4 (0.76-2.53) | 0.28 | 1.05 (0.42-2.6) | 0.91 | 2.58 (0.23-28.7) | 0.44 |
| Wheezing | 0.92 (0.47-1.8) | 0.81 | 0.63 (0.2-1.9) | 0.42 | 5.23 (0.47-58.3) | 0.17 |
| Redness of eyes | 0.7 (0.24-2) | 0.53 | 1.5 (0.42-5.3) | 0.53 | NA |  |
| Decreased urine output | 1.7 (0.36-8.6) | 0.48 | NA |  | **21 (1.7-253)** | **0.017** |
| Vomiting | 1.5 (0.84-2.94) | 0.16 | 0.62 (0.20-1.9) | 0.41 | NA |  |
| **Other pathogens co-occurrence** | |  |  |  |  |  |
| Bacteria | 1 (0.99-1) | 0.4 | 0.6 (0.27-1.32) | 0.2 | NA |  |
| Multiple infection | 1.82 (0.85-3.93) | 0.12 | **6.8 (2.86-16.5)** | **<0.001** | NA |  |
| Viral bacterial infection | **3.3 (1.36-8)** | **0.008** | **3.3 (1.36-8** | **0.008** | NA |  |
| *Staphylococcus pneumoniae* | 0.62 (0.16-2.4) | 0.49 | 0.62 (0.16-2.4) | 0.5 | NA |  |
| *Haemophilus influenzae* | 3.38 (0.86-13.34) | 0.08 | 3.38 (0.85-13.3) | 0.08 | NA |  |
| Bocavirus | 3.81 (0.38-38.2) | 0.25 | 3.81 (0.38-38.2) | 0.25 | NA |  |
| Adenovirus | 5.1 (0.47-55) | 0.17 | 5.12 (0.47-55) | 0.17 | NA |  |
| Influenza virus | 0.94 (0.35-2.5) | 0.9 | NA |  | NA |  |
| Respiratory Syncytial Virus | NA |  | 0.94 (0.35-2.5) | 0.9 | NA |  |

*OR (95% CI): Odds Ratio (95% Confidence Interval); **p values were calculated by bivariate logistic regression; Values in bold indicate statistical significance

**Table S5**: Risk factors for *Streptococcus pneumoniae*, *Haemophilus influenzae* and human parainfluenza virus 3 detection among children with influenza-like illness (ILI)

|  | ***Streptococcus pneumoniae*** | | **Parainfluenza virus 3 (HPIV-3)** | | ***Haemophilus influenzae*** | |
| --- | --- | --- | --- | --- | --- | --- |
|  | **OR (95% CI)*** | **P value**** | **OR (95% CI)** | **P value** | **OR (95% CI)** | **P value** |
| Age | 1 (0.95-1.06) | 0.71 | 0.92 (0.8-1.07) | 0.284 | 1.0 (0.95-1.1) | 0.886 |
| Gender | 1.1 (0.56-2.2) | 0.76 | 0.2 (0.02-1.8) | 0.15 | 1.07 (0.51-2.25) | 0.855 |
| Sickness duration | 0.84 (0.66-1.06) | 0.14 | 0.86 (0.44-1.66) | 0.65 | 1.04 (.85-1.3) | 0.685 |
| Similar sickness at home | 0.66 (0.21-2.07) | 0.47 | 5.36 (0. 88-32.6) | 0.068 | **7 (2-24.03)** | **0.002** |
| Number of people at home | 1.07 (0.93-1.23) | 0.35 | 0.96 (0.66-1.37) | 0.8 | .94 (.8- 1.1) | 0.45 |
| Overcrowding (>6) at home | 0.93 (0.43-2) | 0.86 | 0.56 (0.06-5) | 0.6 | .53 (.21- 1.34) | 0.182 |
| Tuberculosis in family | 0.78 (0.2-3.31) | 0.74 | NA | NA | .33 (.04- 2.8) | 0.31 |
| Indoor air pollution | 1.4 (0.92-2.2) | 0.116 | 1.5 (0.5-4.5) | 0.47 | .66 (.4-1.1) | 0.09 |
| Number of children including the child | 1.3 (0.83-2.07) | 0.23 | 0.96 (0.33-2.8) | 0.94 | 1.3 (.84-2.15) | 0.21 |
| Institutional delivery | 1.4 (0.5-4) | 0.52 | 2.85 (0.36-22.7) | 0.32 | 1.2 (.4-3.6) | 0.750 |
| Caesarean delivery | 0.81 (0.4-1.66) | 0.57 | 2 (0.38-10.3) | 0.41 | .95 (.43-2.1) | 0.9 |
| Premature birth | 2.46 (0.25-24.2) | 0.44 | NA | NA | 2.5 (.34-18.7) | 0.36 |
| Birthweight | 1.1 (0.6-2.31) | 0.647 | 2.4 (0.51-11.6) | 0.26 | .72 (.34-1.5) | 0.4 |
| Low birth weight | 0.7 (0.3-1.65) | 0.426 | NA | NA | 1.2 (0.5-3) | 0.65 |
| Breastfeeding | 1.07 (0.96-1.07) | 0.62 | 1.05 (0.93-1.18) | 0.4 | 1 (.93-1.04) | 0.56 |
| Family income | 1.05 (0.58-1.9) | 0.86 | 0.5 (0.11-2.14) | 0.35 | .7 (.36-1.3) | 0.28 |
| Socioeconomic status | 1.67 (0.9-3.1) | 0.1 | 2.8 (0.7-11.1) | 0.14 | 1.5 (.8- 2.9) | 0.2 |
| **Symptom** |  |  |  |  |  |  |
| Cough | 0.8 (0.33-2) | 0.621 | 1.1 (0.12-10.2) | 0.9 | 1.3 (0.5-3.7) | 0.55 |
| Rhinorrhea | 1.67 (0.61-4.5) | 0.31 | 0.76 (0.08-6.9) | 0.8 | **8.17 (1.05-63.7)** | **0.045** |
| Nasal congestion | **2.7 (1.24-6)** | **0.012** | 1.9 (0.22-17.14) | 0.55 | **6.2 (1.77-21.54)** | **0.004** |
| Sore throat | NA |  | NA |  | NA |  |
| Difficulty breathing | 1.44 (0.53-3.9) | 0.47 | 3.3 (0.568-10.4) | 0.187 | **5.65 (2.02-15.77)** | **0.001** |
| Sneezing | 0.87 (0.44-1.73) | 0.7 | 1.4 (0.28-7.38) | 0.67 | 0.84 (0.39-1.8) | 0.65 |
| Wheezing | 0.68 (0.26-1.8) | 0.44 | **7 (1.31-38)** | **0.023** | 2.6 (0.95-7) | 0.061 |
| Redness of eyes | 0.54 (0.16-1.8) | 0.32 | 2.2 (0.23-20) | 0. 5 | 1.25 (0.35-4.44) | 0.72 |
| Decreased urine output | NA |  | NA |  | 1.23 (0.11-14) | 0.86 |
| Vomiting | 0.62 (0.26-1.5) | 0.28 | 0.83 (0.09-7.44) | 0.87 | 1.72 (0.7-4.2) | 0.23 |
| *Staphylococcus aureus* | 0.8 (0.05-13) | 0.87 | **25.6 (1.4-471)** | **0.03** | NA |  |
| *Streptococcus pneumoniae* | NA |  | 1.63 (0.3-9.2) | 0.58 | 1.64 (0.76-3.5) | 0.2 |
| *Pseudomonas aeruginosa* | 0.80 (0.05-13) | 0.87 | NA |  | 2.5 (0.15-41) | 0.52 |
| *Haemophilus influenzae* | 1.64 (0.76-3.5) | 0.2 | 2.6 (0.5-13.4) | 0.26 | NA |  |
| *Moraxella catarrhalis* | 3.3 (0.36-30.5) | 0.28 | NA |  | 1.67 (0.27-10.44) | 0.58 |
| Parainfluenza virus 1 | 1.6 (0.14-18.26) | 0.7 | NA |  | NA |  |
| Parainfluenza virus 3 | 1.63 (0.29-9.2) | 0.58 | NA |  | 2.6 (0.5-13.4) | 0.26 |
| Bocavirus | 3.3 (0.36-30.5) | 0.29 | NA |  | 0.6 (0.06-5.6) | 0.66 |
| Adenovirus | 2.46 (0.25-24.25) | 0.44 | NA |  | 8 (0.8-78.6) | 0.07 |
| Influenza virus | 2.45 (0.25-24.25) | 0.44 | NA |  | 8 (0.8-78.6) | 0.07 |
| Respiratory Syncytial Virus | 0.62 (0.16-2.4) | 0.49 | NA |  | 3.4 (0.86-13.3) | 0.08 |

*OR (95% CI): Odds Ratio (95% Confidence Interval); **p values were calculated by bivariate logistic regression; Values in bold indicate statistical significance
